# Can patients with low health literacy be identified from routine primary care health records? A cross-sectional and prospective analysis

**DOI:** 10.1186/s12875-019-0994-8

**Published:** 2019-07-18

**Authors:** Paul Campbell, Martyn Lewis, Ying Chen, Rosie J. Lacey, Gillian Rowlands, Joanne Protheroe

**Affiliations:** 1 Arthritis Research UK Primary Care Centre, Research Institute for Primary Care & Health Sciences, Keele University, Staffordshire, ST5 5BG UK; 2 Midlands Partnership NHS Foundation Trust, St Georges’ Hospital, Stafford, ST16 3AG UK; 3 Institute of Health and Society, Newcastle University, Newcastle upon Tyne, NE1 7RU UK

**Keywords:** Health literacy, Primary care, Electronic health records, Musculoskeletal pain

## Abstract

**Background:**

People with low health literacy (HL) are at increased risk of poor health outcomes, and receive less benefit from healthcare services. However, healthcare practitioners can effectively adapt healthcare information if they are aware of their patients’ HL. Measurements are available to assess HL levels but may not be practical for use within primary care settings. New alternative methods based on demographic indicators have been successfully developed, and we aim to test if such methodology can be applied to routinely collected consultation records.

**Methods:**

Secondary analysis was carried out from a recently completed prospective cohort study that investigated a primary care population who had consulted about a musculoskeletal pain problem. Participants completed questionnaires (assessing general health, HL, pain, and demographic information) at baseline and 6 months, with linked data from the participants’ consultation records. The Single Item Literacy Screener was used as a benchmark for HL. We tested the performance of an existing demographic assessment of HL, whether this could be refined/improved further (using questionnaire data), and then test the application in primary care consultation data. Tests included accuracy, sensitivity, specificity, and area under the curve (AUC). Finally, the completed model was tested prospectively using logistic regression producing odds ratios (OR) in the prediction of poor health outcomes (physical health and pain intensity).

**Results:**

In total 1501 participants were included within the analysis and 16.1% were categorised as having low HL. Tests for the existing demographic assessment showed poor performance (AUC 0.52), refinement using additional components derived from the questionnaire improved the model (AUC 0.69), and the final model using data only from consultation data remained improved (AUC 0.64). Tests of this final consultation model in the prediction of outcomes showed those with low HL were 5 times more likely to report poor health (OR 5.1) and almost 4 times more likely to report higher pain intensity (OR 3.9).

**Conclusions:**

This study has shown the feasibility of the assessment of HL using primary care consultation data, and that people indicated as having low HL have poorer health outcomes. Further refinement is now required to increase the accuracy of this method.

## Background

Health literacy (HL) is conceptualised as the measure of “*people’s knowledge, motivation and competencies to access, understand, appraise, and apply health information in order to make judgments and take decisions in everyday life concerning healthcare, disease prevention and health promotion to maintain or improve quality of life during the life course”* [1]). Those with limited HL skills are at higher risk of a range of poor health outcomes [2, 3], receive an inefficient mix of health care services, with care biased towards acute and emergency care rather than planned and preventative care [4–6].

A recent review of evidence within the UK has shown that health materials and health text used to explain information about treatments, self-management, health promotion, disease prevention and health systems are at a significant mismatch with population literacy and numeracy capability [7]. This creates an imbalance; patients at greatest need of such information are least able to benefit from the information, with evidence that those most actively engaged in healthcare decision making tend to be younger, articulate, and of higher socio-economic status [8]. These findings contrast the context of UK government policy to increase patient participation in healthcare, with a particular emphasis on better information to enable an informed choice of available healthcare options [5, 9]. There is therefore a need to identify patients with low HL, and adapt healthcare communication and management accordingly; studies have shown health care practitioners can adapt their communication once aware of a patient’s HL level, and such adaptations have been shown to have patient benefit [4, 10]. In terms of the identification of those with low HL, historically HL was assessed through the creation of specific comprehension tasks often included within large population surveys [8, 11], however concerns arose in terms of the practicality of such measures due to the time required to complete. This led to the development of shorter tests or simpler screening questions, and these methods were proven to have accuracy compared to “Gold Standard” measures (e.g. Newest Vital Sign [12], Single Item Literacy Screener [13]). Nonetheless these measurements still entail engagement, assessment and measurement, which can prove difficult for individuals with low HL, who are much less likely to disclose difficulties in literacy, and less likely to participate in research [14–16]. To overcome this issue, researchers noted consistent high correlations between HL scores and certain demographic measures (e.g. age, sex, ethnicity, years in education) leading to the development of “proxy” measures of HL [11, 17]. An example is the Demographic Assessment of Health Literacy (DAHL) which has been shown to have acceptable approximation to HL assessment [11, 18].

In this study we wish to test whether a similar demographic assessment of HL could be derived from routinely collected information within primary care patient healthcare records, enabling general practitioners (GPs) and other healthcare professionals to be informed of a patient’s likely HL level at the point of consultation. Using an existing cohort, specific objectives are to (i) test the accuracy of a demographic proxy measure of health literacy (based on Hanchate et al’s DAHL [11]) compared to a validated measure of health literacy, (ii) consider additional indicators for this demographic proxy measure that improve accuracy, (iii) identify suitable indicators of the improved proxy measure within primary care electronic health records, and (iv) test this final improved proxy measure in the prediction of poor health outcomes.

## Methods

Secondary analysis was carried out from a recently completed prospective cohort study; Keele Aches and Pains Study (KAPS, for full details of KAPS please see study protocol [19]). The overall aims of KAPS were to further refine and validate a prognostic screening tool to identify patients who have consulted about musculoskeletal pain at risk of poor health outcome at the point of consultation. This dataset was chosen for this study because consultations for musculoskeletal pain conditions are common in primary care (up to 20% of the primary care population will consult about musculoskeletal conditions in a given year [20]), and most often require significant patient/healthcare engagement and self-management over time, therefore potentially placing HL as an important factor for this patient population [21–23]. Furthermore, a recent prospective study, using the KAPS dataset, showed those with low health literacy reported lower levels of physical function and higher levels of pain intensity at 6 months compared to those with adequate levels of health literacy, illustrating the relevance of HL status in this population [24].

Ethical Approval for KAPS was granted by the South East Scotland Research Ethics Committee (REC ref. 14/SS/0083). Participants were informed at the consent stage of KAPS that collected anonymised data may be used within other research studies subject to our Research Institute’s Data Request Procedures and Research Governance Practice.

### Recruitment procedure for KAPS

Briefly, adult patients who visited their GP with one (or more) of the five most common musculoskeletal pain presentations: back, neck, shoulder, knee or multi-site pain (i.e. more than one of the specified sites) were invited to take part. In total 14 primary care practices from throughout the West Midlands and Staffordshire areas of the UK participated. Patients had to be registered at participating primary care practices, be aged 18 years or over, and consult with a musculoskeletal pain presentation. Participants were excluded if there was indication of serious pathology or patient vulnerability (e.g. experienced recent trauma, cognitive impairment, dementia, terminal illness). Individual patients who consulted with back, neck, shoulder, knee or multi-site pain were identified through relevant musculoskeletal symptom and diagnostic ‘Read’ codes. General practitioners within the UK enter medical diagnosis or symptoms using Read codes which are organised into a hierarchical recording system [25]. All eligible patients received a baseline questionnaire shortly after their index consultation. Those patients who returned the questionnaire (*n* = 1890) signified a wish to take part and received further questionnaires at 2 month (*n* = 1428, 75.5%) and 6 month (*n* = 1453, 76.9%) follow up points. In addition, participants were asked to give consent for access their primary care “electronic healthcare records” (EHR) over the time period of the study (1 month prior to index consultation up to 6 months follow up stage). This sub-group (*n* = 1506, 79%) who gave consent for the study team to access their EHRs forms the cohort to be used in this current study.

### Measures

The benchmark assessment of health literacy (HL) was measured at baseline in KAPS using the Single Item Literacy Screener (SILS): “How often do you need to have someone help you when you read instructions on pamphlets, or other written material from your doctor or pharmacy?” [13]. Response options were often, always, sometimes, rarely, never, and the identification of “low” HL was taken by combining groups who had responded “often”, “always” and “sometimes” to create two groups (low and adequate HL) following previous methodology [13, 24].

The components of the proxy demographic assessment of health literacy measure (Cohort DAHL), as generated from the cohort data, followed guidance from Hanchate et al. [11], to include the following risk factors; age (age 70 and above), gender (female), years of schooling completed (< 8 years), and ethnicity (non-European). Self-report measures in the KAPS dataset were: current age, measured from date of birth; gender; number of years in full time education. Ethnicity was not available in the KAPS dataset.

In order to improve this Cohort DAHL a number of additional measures within the KAPS dataset were examined as potentially associated with low HL. These measures were selected based on previous research demonstrating associations with HL [2, 24, 26, 27], and also the likelihood of these measures being available (i.e. either directly or via proxy) within primary care medical records (Table 1). Potential HL indicators explored were; sleep problems, mental health, mobility, comorbidity, social isolation and employment status.

**Table 1:** Potential low health literacy indicators from KAPS cohort and KAPS participant primary care medical records

Domain	Measures within KAPS cohort (self-report)	Example comparative Read codes for use in Electronic Healthcare Records (EHR) analysis^a^
*Age*	Yes	Date of birth
*Gender*	Yes	Gender
*Socioeconomic index*	Number of years in full time education	Area level index of deprivation for each GP practice (provided by the Index of Multiple Deprivation, from the Office of National Statistics, UK [28])
*Ethnicity*	Not recorded	Ethnicity Read codes (e.g. 9i00. White British, 9T1B. South Asian, 13Z69 First Language not English, 9NU.. Need for interpreter)
*Sleep problems*	Jenkins Sleep Questionnaire [29]	Sleep problem consultation Read codes (e.g. Fy0.. Sleep disorders, R0053 Hypersomnia sleep apnoea, 1BX0. - Delayed onset of sleep)
*Mental Health*	SF 36 Mental Component Score [30]	Mental health consultation symptoms and diagnoses (e.g. Eu3.. Mood affective disorders, E1 … Non organic psychosis)
*Mobility*	EQ 5D-5 L Mobility [31]	Measures of mobility and function (e.g. 39... Disability assessment, 39H.. Continence assessment, 395.. Dressing ability)
*Comorbidity*	Presence of other long term medical conditions (e.g. Diabetes, Breathing problems/COPD, Heart problems/High blood pressure, Chronic Fatigue Syndrome/Fibromyalgia, Stress, Anxiety or Depression, or other comorbidity).	Charlson Index of comorbidity covering 17 common comorbidities using BNF prescribing codes [32, 33]
*Social isolation*	Live alone [34]	Social isolation Read codes (e.g. 13HL. Social isolation, 1B1K. Loneliness).
*Employment*	Employment status (working, not working due to health/illness, other, e.g. retired, student, homemaker)	Current employment status (e.g. 13 J7. Unemployed, 9 K8.. Sickness certificate, 13JL. Medical problems at work).
*Consultation/prescription frequency*	Not recorded	Count of consultations and count of prescriptions of the time period of medical record review.

*KAPS* Keele Aches and Pains Study, *BNF* British National Formulary

^a^Full Read code lists available on request

Sleep problems were assessed using the Jenkins Sleep Questionnaire (contains 4 questions about sleep problems over past 4 weeks; “trouble falling asleep”, “waking up several times”, “trouble staying asleep”, and “waking up after usual amount of sleep feeling tired”) [29], participants were classified into two groups based on responding “not at all” or “some nights” compared to those responding on “most nights”, following previous methodology [29, 35]. Mental health was assessed using the SF36 Mental Component Score (full scale score was used) [30], and mobility assessed using the “Mobility” subscale of the EQ. 5D (consisting of 5 response categories) [31]. Comorbidity was assessed by asking participants to indicate the presence of other long term conditions (e.g. diabetes, breathing problems/COPD, heart problems/high blood pressure, chronic fatigue syndrome/fibromyalgia, stress, anxiety or depression, or other comorbidity) and the variable was constructed as “none”, “one or two”, “three or more” comorbid conditions. Social isolation was assessed by the question “do you live alone” with yes/no response categories following previous methodology [34]. Employment status was assessed using the following responses (working, not working due to health reasons, not working other, e.g. retired, student, homemaker).

Read codes were used as the system to capture variables within the electronic healthcare records (EHR) of the KAPS participants corresponding to the questionnaire items. Read codes are the coding system GPs use when entering patient information (e.g. symptoms, diagnosis, prescriptions, demographic and status information) on their computerised systems in the UK [25], and methodology using Read codes forms an acceptable and valid platform for health research [36, 37]. This process involved the search of existing relevant Read code lists (e.g. https://clinicalcodes.rss.mhs.man.ac.uk/, www.keele.ac.uk/mrr/morbiditydefinitions) as well as searching the NHS clinical terminology browser (provides Read code lists for diagnoses, symptoms, and status via a search facility). Read codes were used that were directly relevant (e.g. mental health, comorbidity), and Read codes were also added that may be proxy markers of components that would be less likely to be recorded within medical records (e.g. low area level deprivation level as a proxy marker of less years of formal education). Each Read code list was then reviewed by the research team inclusive of a GP (JP) for consensus (see Table 1 for examples, full list of Read codes available on request).

Finally, two outcome measures were chosen to establish the ability of the proxy demographic assessment of health literacy derived from primary care medical records (i.e. the final model derived from the Cohort DAHL using only medical record information). This medical record DAHL (termed General Practice Health Literacy Assessment or “GP-HLA”) will be used to predict patient self-report health outcomes at 6 month follow up. The first measure was the Short Form-36 (SF-36) Physical Component Score (PCS) which is a general measure of health related quality of life and physical function [30]; for analysis a dichotomy (recovery/non recovery) was created based on a PCS cut off criteria (39.61), as used in KAPS [19], and derived from an independent similar musculoskeletal pain cohort study [38]. The second measure reflected a relevant musculoskeletal pain outcome for this cohort; current pain intensity was measured on a 0–10 numerical rating scale, 0 indicating no pain, 10 indicating pain as bad as it could be, dichotomised using a validated cut point (score above 4) to indicate non recovery [39–41].

### Statistical analysis

Analysis followed stages that correspond to the study objectives. Stage i) tested the accuracy and validity (predictive, discriminant) of a derived measure of HL (based on Hanchate’s DAHL [11]) from the cohort data termed “Cohort DAHL” (comprising age (> 70), sex (Female), and education level (Hanchate et al. recommend < 8 years’ education; however the variable within KAPS asked age when person left full time education and so a calculation was made, based on start of school at age 5, of the cut point at age 12. Inspection of the data revealed only 0.7% had left school at age 12, and so this age range was raised to age 14 to give greater scope for analysis at 5.9%). This “Cohort DAHL” was tested in comparison to the SILS measure (dichotomous category of low and adequate HL). Tests chosen included sensitivity and specificity (the percentage of actual positives correctly identified, and the percentage of negatives correctly identified respectively), positive and negative predictive values (the percentage probability of a true positive and probability of a true negative respectively), area under the curve (value of discrimination), logistic regression (test of association), and variance accounted (proportion of the variance in an outcome that is explained by the factors within a model). Stage i) analysis reports sensitivity and specificity, positive and negative predictive values (PPV, NPV), area under the curve value (AUC), Logistic Regression Odds Ratio with 95% confidence intervals (OR 95% CI), and the variance accounted R^2^ (Nagelkerke) for this Cohort DAHL based on those who fulfilled all criteria. Stage ii) iteratively tested improvements to this Cohort DAHL (Improved Cohort DAHL) by evaluating a range of items available within the dataset in comparison to the SILS categories (Table 1). Items were selected for inclusion based on their (per item univariate analysis) AUC score > 0.55 to indicate above chance accuracy [42], and/or presence of statistical significance within a univariate logistic regression test with the SILS measure. Those items that fulfilled the univariate inclusion criteria were added to the Cohort DAHL within a final combined (all items together) multivariate logistic regression test (at this point redundancy checks were made and all non-significant variables removed). Because the addition of new variables without a predetermined dichotomous criterion were added (e.g. full scale scores) the predicted probabilities for each participant were produced from the final multivariate logistic regression model for the final Cohort DAHL and a dichotomy created using a cut off point that matched the proportion of those with low HL within the cohort (i.e. to represent the expected proportion of those with low HL), to create categories of low and high predicted probability of low HL. This dichotomous variable was used to produce sensitivity and specificity, positive and negative predictive values (PPV, NPV) and AUC discrimination score. Stage iii) evaluated potential proxy markers of all Improved Cohort DAHL items within EHR Read codes (see Table 1). Read code based variables were created within the consultation EHR of the KAPS participants corresponding to the final Cohort DAHL, this then produced a General Practice Health Literacy Assessment tool “GP-HLA” (i.e. a DAHL consisting only of information derived from routine primary care EHR). The accuracy and validity of the new GP-HLA in comparison to the SILS measure was examined using the same tests as performed at Stage i) and Stage ii). Finally, Stage iv) involved exploratory analysis to test prospectively the final GP-HLA in the prediction of self-report poor health outcomes (Physical Component Score, Pain Intensity) using logistic regression analysis producing ORs and 95 CIs. Complete case analysis was carried out due to the low level (≤ 5%) of missing data within the KAPS questionnaire responses. All analysis was carried out on SPSS (version 24), and the Medcalc diagnostic test evaluation calculator was used to produce 2 × 2 table sensitivity and specificity and PPV/NPV values (https://www.medcalc.org/calc/diagnostic_test.php, accessed December 2018).

## Results

Baseline characteristics are outlined in Tables 2 and 3 and are presented by category of HL (adequate or low) and by the full cohort. In total 16.1% of the cohort (*n* = 241) were classified as low HL using the SILS measure, and inspection of the comparison between those with adequate and low HL in Table 2 shows those within the low HL category have; older age (not significant), more females (not significant), lower age at leaving full time education, higher level of sleep problems, lower mental health score, higher presence of mobility issues, increased comorbidity, more likely to live alone, and a greater percentage of being off work due to health reasons.

**Table 2:** Descriptive characteristics of KAPS cohort (questionnaire data)

Measure	SILS categorisation (based on the need for help in reading health-related materials)		Total Mean (SD) or %
	*Adequate health literacy - Mean (SD) or %*	*Low health literacy - Mean (SD) or %*	
SILS % (n)	83.9% (*n* = 1260)	16.1% (*n* = 241)	*n* = 1501
Age (years)	57.8 (15.6)	59.1 (16.7)	58.1 (15.8)
Gender (Female)	57.8%	58.5%	57.9%
Age left school (years old)	16.0 (1.2)	15.3 (1.7)^a^	15.9 (1.3)
Sleep problems	62.7%	76.2%^a^	64.8%
Mental component score (SF36)	44.5 (12.5)	33.9 (12.5)^a^	43.7 (13.2)
EQ 5D Mobility	2.1 (1.0)	2.9 (1.1)^a^	2.2 (1.1)
Comorbidity (KAPS, 3 or more)	8.4%	22.8%^a^	10.7%
Live alone (social isolation)	19.5%	28.3%^a^	20.9%
Employment (off work due to health reasons)	8.8%	29.9%^a^	12.2%

^a^Significant test between adequate and low health literacy, *p* value< 0.05 (two sided t-test or X^2^ test used where appropriate)

**Table 3:** Descriptive characteristics of the KAPS cohort (EHR data)

Measure	SILS categorisation (based on the need for help in reading health-related materials)		Total Mean (SD) or %
	*Adequate health literacy - Mean (SD) or %*	*Low health literacy - Mean (SD) or %*	
IMD deprivation score^a^	5.94 (3.2)	5.11 (3.2)^b^	5.81 (3.2)
Monthly consultation frequency	1.73 (1.3)	2.39 (1.8)^b^	1.84 (1.4)
Monthly prescription frequency	5.29 (6.1)	13.43 (18.9)^b^	6.60 (9.9)
Comorbidity (Charlson index score [32])	8.26 (6.3)	13.34 (8.6)^b^	9.08 (6.9)
Mental health consultation	23.5%	40.7%^b^	26.2%
Sleep problem consultation	7.8%	18.3%^b^	9.4%

*SD* standard deviation

^a^IMD mean decile score, lower score indicates increased deprivation

^b^Significant test between adequate and low health literacy, *p* value< 0.05 (two sided t-test or X^2^ test used where appropriate)

### Analysis stages i) and ii): testing the accuracy and validity of derived demographic assessments of health literacy (DAHL) and improvements to the DAHL within the KAPS cohort data

Results are shown in Table 4 (1st and 2nd rows). The stage i) model (age, gender, left full time education at age 14 or under) showed an overall low performance across the parameters for the Cohort DAHL. Stage ii) analysis considered the components of the existing Cohort DAHL (stage i) and additional components to improve performance of the Cohort DAHL (as shown in Table 1). Individual univariate tests (logistic regression and AUC with 95% confidence intervals) showed that age and gender had no relation to low health literacy; however, leaving full time education at age 14 or under (OR 4.27 95% CI 2.7 to 6.7, AUC 0.56 95% CI 0.51 to 0.60), sleep problems (OR 1.90 95% CI 1.4 to 2.6, AUC 0.57 95% CI 0.53 to 0.61), mental health (OR 0.93 95% CI 0.92 to 0.94, AUC 0.75 95% CI 0.71 to 0.78), mobility (OR 1.97 95% CI 1.7 to 2.3, AUC 0.69 95% CI 0.65 to 0.73), comorbidity (presence of 3 or more conditions as test category against reference of none, OR 4.91 95% CI 3.1 to 7.7, AUC 0.62 95% CI 0.58 to 0.66), social isolation (OR 1.64 95% CI 1.2 to 2.2, AUC 0.54 95% CI 0.50 to 0.59), and being off work due to health reasons (OR 8.69 95% CI 5.6 to 13.5, AUC 0.59 95% CI 0.55 to 0.62) were all selected. The multivariate model including all these significant factors showed an increase in variance explained from 1% in the stage i) model to 27.2% at stage ii), but also revealed non significance for social isolation and sleep problems, and so these items were removed. The final multivariate model (including age left school, comorbidity, employment, mental health, and mobility) showed a variance explained of 26.9%. Predicted probabilities were derived from this final model and an 83.9 percentile cut point was calculated (based on a population level of low HL at 16.1%) to create two categories (low and high predicted probability of low HL). These groupings were used to produce results on all testing parameters in Table 4 (row 2), and show slightly higher sensitivity, higher specificity, much improved PPV, decreased NPV, improved AUC, improved predictive accuracy, and improved explained variance compared to the stage i) model.

**Table 4:** Accuracy, discriminatory and predictive tests for the cohort DAHL, improved cohort DAHL, and GP-HLA assessments of health literacy

Analysis stage^a^	Sensitivity (95% CI)	Specificity (95%CI)	PPV (95% CI)	NPV (95% CI)	AUC (95% CI)	OR (95% CI)	Percentage variance (R^2^ × 100)
Stage i) Application of Cohort DAHL	36.4% (20.4 to 54.9)	84.7% (82.7 to 86.5)	5.2% (3.3 to 8.0)	98.3% (97.8 to 98.7)	0.52 (0.48 to 0.56)	3.16 (1.5 to 6.5)	1%
Stage ii) Application of Improved Cohort DAHL	46.8% (40.2 to 53.7)	90.3% (88.5 to 92.0)	48.1% 42.5 to 53.8)	89.9% (88.6 to 90.9)	0.69 (0.65 to 0.73)	8.26 (5.9 to 11.5)	18.4%
Stage iv) Application of the GP-HLA	40.1% (33.9 to 46.6)	88.6% (86.7 to 90.3)	40.3% (35.1 to 45.6)	88.5% (87.4 to 89.5)	0.64 (0.60 to 0.69)	5.18 (3.8 to 7.1)	11.2%

*PPV* Positive predicted Value, *NPV* Negative Predicted Value, *AUC* Area Under the Curve, *OR* Odds Ratio, *CI* Confidence Interval, *R*^*2*^ Nagelkerke value, *DAHL* demographic assessment of health literacy

^a^Stage i variables (age, gender, age left full time education), Stage ii variables (age left full time education, comorbidity, employment, mental health, mobility), Stage iv variables (prescription frequency, comorbidity, deprivation, mental health)

### Analysis stage iii) evaluation of cohort DAHL markers within electronic health record (EHR) read codes and descriptive of GP-HLA components

Inspection of the EHR data revealed a number of status variables (ethnicity, mobility, employment, and social isolation) were poorly recorded (> 98% data not recorded), therefore these variables were not used within the analysis. Descriptive characteristics for the EHR variables used within the analysis are presented in Table 3. Results of these characteristics stratified by the SILS health literacy categorisations show differences between the HL groups, with those with low HL having significantly higher deprivation scores, higher levels of consultation and prescription frequency, higher levels of comorbidity, and a greater prevalence of mental health and sleep problem consultations, however no statistical differences were shown for age and gender.

### Analysis stage iv) testing the accuracy and validity of the GP health literacy assessment (GP-HLA) derived from patient electronic health records with the assessment of health literacy from the KAPS cohort

Testing the individual components identified within stage iii) within univariate logistic regression tests showed all variables were individually significantly associated with the SILS HL categorisations. Testing all the components within a multivariate model showed deprivation, prescription frequency (per month), comorbidity, and mental health were significant, with consultation frequency (per month) and presence of sleep problems as not significant. A refined model (Table 4, row 3) including the significant variables and adjusting for age and gender was tested, all variables (apart from age and gender) retained significance and the percentage variance explained was 16%. Application of the 83.9 cut point to the predicted probabilities showed a final GP-HLA model that accounts for 11.2% variance and shows higher sensitivity, higher specificity, higher PPV, lower NPV, improved AUC, improved predictive ability and increased % variance of the model at stage i).

### Analysis stage v) testing prospectively the predictive ability of the GP-HLA for poor self-report health outcomes

Analysis of the GP-HLA using logistic regression in the prospective prediction of poor outcomes (pain intensity > 4, physical component score > 39.61) showed that those who were included within the GP-HLA categorisation of low HL were at almost 4 times more likely to experience pain that does not recover (OR 3.91 95%CI 2.9 to 5.3), and over 5 times the odds (OR 5.07 95% CI 3.5 to 7.3) to continue to have poor physical health at 6 months.

## Discussion

This study has demonstrated the feasibility of deriving an approximation of a patient’s HL status directly from routine primary care health record data. The results show a significant improvement in the accuracy, discriminative, and predictive ability from a currently used demographic health literacy assessment (DAHL), and has also demonstrated prospectively that assignment of low HL status, as identified via primary care health records, is predictive of poor health outcomes in this cohort.

### Comparison with previous literature

In this current cohort just over 16% of the population were defined as low HL using the categorisations “always, often, sometimes” from the SILS measure following previous methodology [13, 24]. This percentage of low HL is similar to Morris et al. [13] (who used the S-TOFHLA and report 17%) within their primary care population who were participating in a diabetes RCT. However, the figure of 16% is consistently lower than other estimates, for example a review of 85 studies estimate a prevalence of 26% [17], a recent survey assessment of directly measured HL within an urban area (Stoke-on-Trent) reports a level of 28.5% [43], and an assessment of literacy competency reports at 43% using a UK wide purposeful sample of the working population [7]. These differences may be explained by the differences in the assessment of HL, for example Protheroe et al. [43] used the Newest Vital Sign as the assessment of HL, and Rowlands et al. [7] used multiple domains for assessment (e.g. health promotion, disease prevention, managing illness, health service navigation) both of which are assessments of function and ability compared to the self-report SILS. Another key reason for the difference is the target populations used, these higher levels of low HL are typically derived from general population cohorts, whereas this current study (and Morris et al. [13]) focused on a primary care population. This variation may reflect a generally lower engagement of those with low health literacy in non HL focused research (particularly research such as the KAPS study which involved a lengthy written questionnaire), as compared to specific HL focused studies where strategies for recruitment of this population would be more likely [44], for example both Protheroe et al. [43] and Rowlands et al. [7], employed face to face interview assessments in representative population samples. Future validation studies will be required to test whether the method to identify those with low HL developed here, accurately represents the actual proportion of those with low HL who engage within primary care.

Examination of the results on the application of the Cohort DAHL (as prescribed by Hanchate et al. [11]) show an overall poor performance on accuracy, discrimination and predictive ability with a particularly low variance explained (1%) and just above chance level AUC (0.52). A recent study that used similar components to Hanchate et al. (e.g. qualifications, age, language, deprivation) in the prediction of health literacy competency found adequate discrimination (AUC 0.75), however the proportion of those judged as low competency was much higher (43%) and the assessment of HL was much more comprehensive and therefore more likely to have greater sensitivity [18]. Comparison of the components of the DAHL within this current study and the original Hanchate et al. study show some key differences. For example, this study was unable to include ethnicity/race due to non-collection of this data within the KAPS questionnaire (and subsequently poor recording within EHR data) and this may have contributed to the lower performance; Hanchate et al. reports 76% as white within their population, whilst a recent HL survey (carried out within a similar geographical area to this current study, [43] reports 91% white, therefore demonstrating the application of race/ethnicity can be population specific. Another key difference is in the categorisation of education level (the most significant driver of the assessment of low HL within the Hanchate et al. paper and in this current cohort analysis); within the Hanchate et al. study 17 to 19% reported years of schooling below 8 years, however this study reported only 0.7% who fit this criterion, and adjustments were required to increase this age range by 2 years to enable a feasible analysis. Another important factor is age at participation, the Hanchate et al. study included those aged 65 and above (therefore inflating the population above the age of 70), whereas this current study included anyone aged 18 and above. Inclusion of younger people would have increased the length of full time education compared to the Hanchate cohort. School leaving age has changed over time in the UK, for example the formal school leaving age moved from age 14 to age 15 in 1947 and from 15 to 16 in 1972, therefore cohort effects are present and consideration should be taken to the historical context when measures of formal education are used as this will change as policy changes [45, 46]. Overall comparison between the application of the DAHL from the Hanchate et al. study and this current study is difficult due to these key differences, and this conclusion falls within a general issue and criticism of consistency across different populations in the assessment of HL [47].

### Strengths and limitations

The inclusion of additional components (sleep problems, mental health, mobility, comorbidity, social isolation, employment status) to the DAHL (i.e. Improved Cohort DAHL) showed significant improvements in relation to the SILS category of low HL (1% variance improved to 27%, AUC improved from 0.52 to 0.69). These components were chosen based on previous literature demonstrating associations, and specifically as items that could be captured (either directly or by proxy) within EHR data. It is clear that the original components of the DAHL could be seen as causal or as moderating factors (age, gender, years of schooling, race) whereas these additional components would be viewed as mediators or perhaps consequences of low HL, and therefore should not be considered as part of a model to “explain” the development of low HL, but can be seen as components that can assist in the identification of those with low HL (the key purpose of this study). The success of the identification of components with EHR data was mixed, there was only a short window of time for medical record review in this study (7 months), and notably the capture of status variables such as ethnicity/race, social isolation, mobility/disability, and employment status was not possible (low recording within EHR) and this reflects a general criticism of EHR data where information on lifestyle and health behaviours are not well recorded [48]. In a recent study that used population level demographic information derived from EHR to identify population level expected proportions of low HL reports on potential feasibility, though key information such as ethnicity needs to be more completely recorded [49]. Furthermore, as EHR does not routinely record information on education level, this study used an approximation related to deprivation levels (deprivation from IMD index for participating GP practices). There is robust evidence that years of schooling is related to deprivation and so a valid inclusion of deprivation as a proxy measure of education in this study [50, 51], however further refinements in this proxy measure, such as the patient’s actual household postcode (to give indication of household level of deprivation) may have led to greater discriminatory power. Nonetheless the GP-HLA was shown to have greater discriminative and predictive performance compared to the initial cohort DAHL, with a substantial improvement of positive predictive value (probability that low HL is identified as predicted, from 5.2 to 40.3%), with only a small reduction in negative predictive value (probability that low HL is not present when predicted, from 98.3 to 88.5%) with increased variance (1 to 11%) and improved AUC (0.52 to 0.64). Furthermore, the categories created within the GP-HLA were then shown to successfully prospectively predict poor health outcomes at 6 months follow up, demonstrating a useful platform to understand the long term outcomes for those with low HL within EHR data. However, the AUC value of 0.64 for the GP-HLA is considered less than acceptable as a value for discrimination (threshold of 0.7 is recommended [52]), and therefore further improvement is required. Aside from the specific points discussed above, the overall key strengths of this study are; the use of a large prospective primary care consultation cohort with linked patient EHR, the ability to include a range of measures (and proxy measures within EHR) that improved the overall performance of a DAHL, and the ability to prospectively test the relationship of low HL status to health related outcomes. General limitations include the inclusion of a cohort restricted to those who had consulted for musculoskeletal pain, whilst this is a common presentation within primary care populations, there is a need to replicate the results within a full primary care consulting population. A number of potential measures were identified that were used to improve the DAHL model (cohort DAHL and GP-HLA), however many more indicators are likely to exist and further research is required to identify these, especially within EHR data. Whilst the predictive ability of the GP-HLA is interesting (prediction of self-report poor health outcomes), the components used to predict (specifically measures of increased healthcare use such as prescription frequency and comorbidity) are in effect measures of poor health also (and so increase autocorrelation effects), and therefore further refinements are required specifically within EHR data to determine appropriate and independent health related outcomes. A further limitation was on the analysis approach. For pragmatic reasons (statistical power) the full cohort was used to both test the validity of the existing DAHL as well as develop improvements to that DAHL. However guidance from “Transparent Reporting of a multivariable prediction model for Individual Prognosis of Diagnosis” TRIPOD, http://www.tripod-statement.org/TRIPOD/TRIPOD-Checklists) suggests that such approaches can lead to “overfitting” or increased confidence in the fit reported. Ideally two cohorts should have been used for this purpose, however in mitigation whilst validation and development for the “DAHL” has taken place within the same cohort, the final model (GP-HLA) utilises measures not connected to the existing or developed “DAHL” and therefore has a greater level of independence of model fit. Finally, a key limitation is the potential restrictiveness of EHR data, as outlined above, aspects such as lifestyle indicators (e.g. employment status, deprivation, healthy behaviours) can be poorly recorded [48], and specific questions central to the calculation of health literacy (e.g. years of education) are not typically recorded. However, this study has demonstrated a useful platform that could be used to identify those who are likely to have low HL and therefore may benefit from changes in primary care management and possibly signposting to other interventions. This information (provided from the patient’s EHR) could be augmented further within the consultation by gathering quick additional confirmatory information in this identified population (e.g. asking patient about their years in education).

### Clinical relevance

The identification of low HL within primary care is important, the consultation is a central place where vital healthcare information (self-management guidance, prescription information, awareness of future health risk) is given, and studies on consultation recollection have shown that patients only retain approximately about one half of this information within a consultation [53]. However, research has shown that GPs, when the HL status of their patients is known to them, change and adapt their consultation style to impart advice and guidance corresponding to the level of understanding of the patient [4], and such adaptations have been shown to reduce emergency department admissions and reduce hospitalisations, reduce disease impact, and increase adherence to medical treatment routines and disease management [44, 54]. This current study has demonstrated the feasibility of a method to identify patients who may have low HL solely from the information already retained by GPs within the EHR system. Future studies are now required to test this model within a general consulting primary care population (as refinements in this population may improve the model and would increase generalisability) and from that interventions could then formulated (e.g. use of techniques such as “teach back”, less use of medical jargon, focus on key points, use of visual aids, ensure written information is appropriate [55]), and evaluated to test whether if changes in communication lead to the improvement of healthcare outcomes for patients within this population.

## Conclusion

In conclusion this study has demonstrated the feasibility of a method to identify those with low HL within a primary care population and demonstrated that such individuals are at higher risk of poor health outcomes. Such information is clinically useful to primary care practitioners and further research is now required to test whether information on a patient’s HL status can lead to improved patient health outcomes.

## Data Availability

The datasets generated and/or analysed during the current study are not publicly available as they refer to individual patient consultation records. However summarised data are available from the corresponding author on reasonable request.

## Abbreviations

AUC: Area under the curve
COPD: Chronic obstructive pulmonary disease
DAHL: Demographic assessment of health literacy
EHR: Electronic health records
GP: General practice/Practitioner
GP-HLA: General Practice Health Literacy Assessment
HL: Health literacy
IMD: Index of Multiple Deprivation
KAPS: Keele Aches and Pains Study
NPV: Negative predictive value
OR: Odds Ratio
PCS: Physical Component Score
PPV: Positive predictive value
SILS: Single Item Literacy Screener
